# Comparison of MR-PWI quantitative and semi-quantitative parameters for the evaluation of liver fibrosis

**DOI:** 10.1186/s12880-020-00539-3

**Published:** 2021-01-06

**Authors:** Ke Ding, Manrong Liu, Xue Wei, Ruisui Huang, Jiong Chen, Shanjin Lu, Dacheng Wang, Wei Lu

**Affiliations:** 1 Department of Radiology, The Third Affiliated Hospital of Guangxi Medical University, No. 13, Dancun Road, Nanning, 530031 China; 2 Department of Ultrasound, The Third Affiliated Hospital of Guangxi Medical University, Nanning, 530031 China; 3 Department of Pathology, The Third Affiliated Hospital of Guangxi Medical University, Nanning, 530031 China

**Keywords:** Liver fibrosis, MR-PWI, Quantitative and semi-quantitative parameters, Liver double blood supply model, Cynomolgus monkeys

## Abstract

**Background:**

To evaluate different stages of liver fibrosis in cynomolgus monkeys by comparing magnetic resonance-perfusion weighted imaging (MR-PWI) quantitative and semi-quantitative parameters, and confirm the best detection indicators for diagnosis of liver fibrosis.

**Methods:**

A liver fibrosis model of different stages (S0–S4) was established in cynomolgus monkeys. The changes in MR-PWI quantitative and semi-quantitative parameters with the progression of liver fibrosis were investigated.

**Results:**

MR-PWI quantitative parameters gradually decreased with the progression of liver fibrosis. Hepatic arterial perfusion index (HPI) was found to increase with the progression of liver fibrosis and significant differences of HPI between each group were observed. There was a highly positive correlation between HPI and the stages of liver fibrosis. Receiver operating characteristic (ROC) curve analysis showed that HPI had the highest efficacy of the MR-PWI quantitative parameters for the diagnosis of liver fibrosis. The MR-PW semi-quantitative parameters gradually reduced with the progression of liver fibrosis, and the differences were statistically significant between stages S3–S4 and S0–S2. Time to peak (TPP) gradually extended and showed a positive correlation with the stages of liver fibrosis. TTP had the highest efficacy of the semi-quantitative parameters for diagnosis of liver fibrosis.

**Conclusions:**

Both the MR-PWI quantitative and semi-quantitative parameters of the liver fibrosis model in cynomolgus monkeys varied at different stages of liver fibrosis, and HPI and TTP were the best detection indices for quantitative and semi-quantitative evaluation of liver fibrosis, respectively.

## Background

Liver fibrosis is a key pathology in the progression of many chronic liver diseases [1], which is a reversible process that can be effectively mitigated and even cured [2, 3]. However, if no effective treatments are conducted, it can develop into liver cirrhosis and even cancer [4–6]. Thus, the early diagnosis of liver fibrosis is crucial to provide some useful information for treatment selection. Liver biopsy has been considered as the “gold standard” for clinical diagnosis of liver fibrosis. However, this method often fails to achieve the dynamic progression monitoring and observations of therapeutic efficacy due to its invasiveness [7–9]. In this regard, the exploration of non-invasive detection technologies for the early diagnosis of liver fibrosis is highly desired. Some non-invasive serological and imaging evaluation methods, such as multiple serum indexes-based liver fibrosis scoring model [10–13], transient elastograhy (TE) [14, 15], acoustic radiation force impulse imaging (ARFI) [16, 17], and magnetic resonance elastography (MRE) [18–20] have shown great promise for the diagnosis of liver fibrosis stages.

Magnetic resonance imaging (MRI), a powerful and noninvasive imaging technique with high spatial resolution and tomographic capabilities, has displayed great promise for the diagnosis of liver fibrosis in the clinic [11, 19, 21]. In particular, magnetic resonance-perfusion weighted imaging (MR-PWI) is a functional imaging technique that can simultaneously reflect the changes of tissue organ morphology and blood perfusion information [22, 23]. MR-PWI has demonstrated unique advantages for the detection of various diseases, while its feasibility for the diagnosis of liver fibrosis has been poorly reported. In addition, animal models are crucial for the evaluation of liver fibrosis using MR-PWI. In most previous studies, lower animals are often used for research, but the experimental results are greatly affected by their individual differences [24]. In contrast, higher animals, such as cynomolgus monkeys have been considered better candidates for studies of human life because their physiologies are extremely similar to those of humans [25–27]. However, the use of a liver fibrosis model in cynomolgus monkeys for MR-PWI studies has not been explored so far.

In this study, a liver fibrosis model of different stages in cynomolgus monkeys was established, and MR-PWI was conducted to evaluate liver fibrosis by comparing MR-PWI quantitative and semi-quantitative parameters. The dynamic changes of these parameters with the progression of liver fibrosis were compared and analyzed to identify the best detection indices for the diagnosis of liver fibrosis. This study thus provides a promising tool for the noninvasive diagnosis of liver fibrosis.

## Methods

### Experimental animals

All animal experiments were reviewed and approved by the medical ethics committee and experimental animal ethics committee of our university. Healthy male cynomolgus monkeys (7 years old, 6.0–7.0 kg body weight) were purchased from the Crab-eating Macaque Breeding Base and Laboratory Animal Center (Guangxi, China). All animals were kept in a clean air-conditioned laboratory room at 25 °C with 50–70% air humidity.

### Materials and instruments

Carbon tetrachloride (CCl_4_, reagent grade, content ≥ 99.5%) and anhydrous ethanol were purchased from Xilong Chemical Co., Ltd. (Guangdong, China). A liver biopsy gun equipped with a semi-automatic biopsy needle (18G L-130 mm) was purchased from TSK Corporation (Tosoh, Tokyo, Japan).

### Establishment of liver fibrosis models

Cynomolgus monkeys were normally fed in a clean air-conditioned laboratory room at 25 °C with 50–70% air humidity for 1 week after purchase. CCl_4_ solution (400 mL/L dilution in olive oil) was subcutaneously injected into each macaque (1 mL/kg body weight) twice a week. Meanwhile, the animals were fed with a high-fat diet supplemented with about 35% cholesterol. The ethanol solution (10% in water) was used as the only drink. At 0, 4, 8, 12, and 16 weeks post-CCl_4_ injection, the cynomolgus monkeys were anesthetized via intramuscular injection of ketamine hydrochloride solution (10 mg/kg body weight). Then, B-ultrasound imaging-guided needle biopsy in the right posterior lobe of the liver was conducted. The length of the specimen was 1.5–2.0 cm. After 20 weeks, the cynomolgus monkeys were anesthetized via intramuscular injection of ketamine hydrochloride solution (10 mg/kg body weight) and then the monkeys were sacrificed through an intravenous injection of air via the ear vein. Liver specimens (1.5 × 1.0 × 0.3 cm) from each macaque were collected for pathological examination.

### Histological analysis

The collected liver specimens were fixed with 4% paraformaldehyde at room temperature for two days, dehydrated in ethanol solution, embedded in paraffin, and then cut into 8-μm sections. Hematoxylin–eosin (HE) and Masson trichrome staining of liver sections was conducted according to the standard protocols. The pathological stages of liver fibrosis were divided into five stages (S0–S4) according to the following criteria as previously reported [28]: S0 (no liver fibrosis), S1 (fibrosis enlargement in the portal area), S2 (fibrosis around the portal area with the formation of a small amount of fibrous septum), S3 (formation of fibrous septum with lobular structure disorder, no cirrhosis), and S4 (early cirrhosis). Among these stages, the S1, S2, and S3–S4 stages were categorized as mild, moderate, and severe liver fibrosis, respectively. In addition, they were also divided by pathological grade (G0-G4) according to the degree of inflammatory activity of the liver tissue.

### MR-PWI imaging and post-processing

**Fig. 1:**
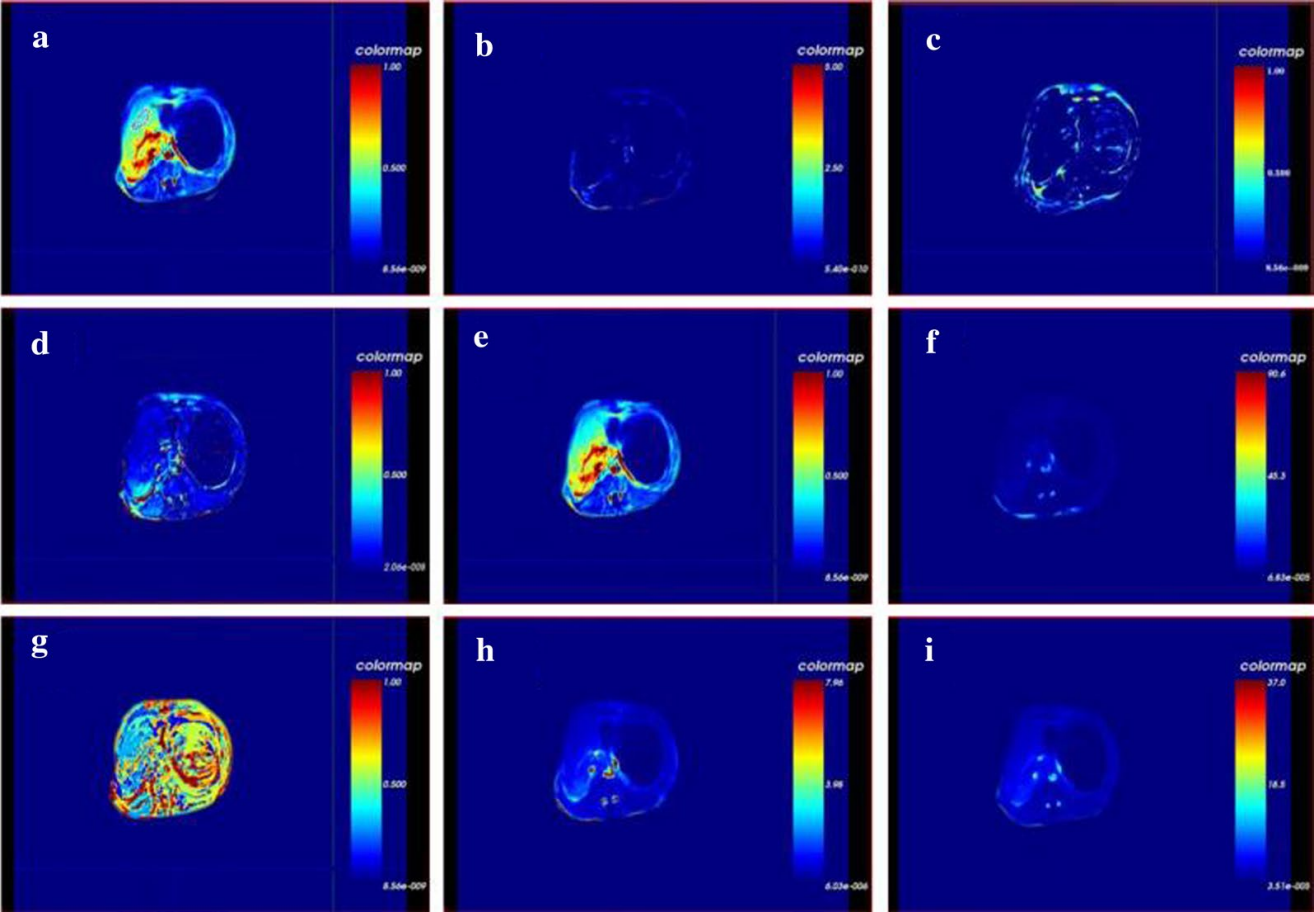
Pseudo-color image of liver perfusion in cynomolgus monkey liver fibrosis model: ROI (**a**), Ktrans (**b**), Kep (**c**), Ve (**d**), Vp (**e**), HPI (**f**), TTP (**g**), MAX conc (**h**) and AUC (**i**)

Cynomolgus monkeys were anesthetized via intramuscular injection of ketamine hydrochloride solution (10 mg/kg body weight). MR-PWI imaging of cynomolgus monkeys was conducted before and after 4, 8, 12, 16, and 20 weeks of the establishment of liver fibrosis models. A Verio 3.0 T superconducting MR scanner (Siemens Company, German) equipped with 8-channel body phased surface coils was used. DCE-MR perfusion imaging was conducted after the completion of a conventional sequence plain scan. Gadolinium-diethylenetriamine penta-acetic acid (Gd-DTPA), a contrast agent for MR imaging was intravenously injected into each macaque using high-pressure syringes at a rate of 1 mL/s (the total volume of 5 mL). A 3D-flash scanning sequence was used for imaging with the following scanning parameters: excitation time = 1, TR = 4.15 ms, TE = 1.32 ms, FOV = 280 × 210 mm^2^, matrix = 156 × 256, thickness = 3 mm, layer interval = 2 mm, image resolution = 1.4 × 1.1 × 3.0 mm^3^. T_1_-weighted MR images were obtained via turning angles of 3°, 6°, 9°, 12°, and 15°, respectively. Then a 9° flip angle was used to complete 45 phase dynamic enhancement scanning (acquisition time = 1, scanning time = 356 s). After the examination, PWI analysis software (Omni-Kinetics Version V2.10) was used for post-processing of the collected images and measurement of parameters. For each case, the registered DICOM data was directly imported into the PWI analysis software, under the exchange liver double blood supply model, region of interest (ROI) was drawn on the abdominal aorta and the portal vein to form the gadolinium concentration–time curve. Then the ROI of liver tissue was drawn, and the software automatically produced a pseudo-color map of the entire liver perfusion, and the quantitative and semi-quantitative parameters of ROI (Fig. 1). Quantitative parameters dependent on the gadolinium concentration–time curve are calculated via the pharmacokinetic model. The quantitative parameters included Ktrans (endothelial transfer constant, rate of blood leakage to extravascular extracellular space [EES]), kep (reflux rate, rate of blood seeping back from the EES), Ve (fractional EES volume, contrast agent extravascular extracellular fluid gap volume, Ve = Ktrans/kep), Vp (fractional plasma volume, contrast agent plasma volume fraction), and HPI (hepatic arterial perfusion index, hepatic artery blood supply ratio). Semi-quantitative parameters describe the shape and composition of the signal intensity-time curve of the ROI. The semi-quantitative parameters included TTP (time to peak), maximum concentration (MAX conc.), maximum slope (MAX slope), and area under the concentration–time curve (AUC).

### Statistical analysis

The experimental animals that died during modeling and failed to develop into early liver cirrhosis were excluded. SPSS 20.0 statistical software was used to conduct the statistical analysis of MR-PWI detection indices for 15 cynomolgus monkeys with complete development of liver fibrosis (S0–S4). The experimental data are expressed as “mean ± standard deviation (SD)”. The comparison of each study index was performed using an incompatibility group design (random area group design) analysis of variance, and the comparison between groups was performed using an SNK q test. Spearman rank correlation analysis was used to determine the correlation between PWI indices and the stages of liver fibrosis. Receiver operating characteristic (ROC) curves were used to analyze and evaluate the diagnostic efficacy of each index in early cirrhosis. P values < 0.05 were considered statistically significant.

## Results

### Histological analysis of liver fibrosis models in monkeys

**Fig. 2:**
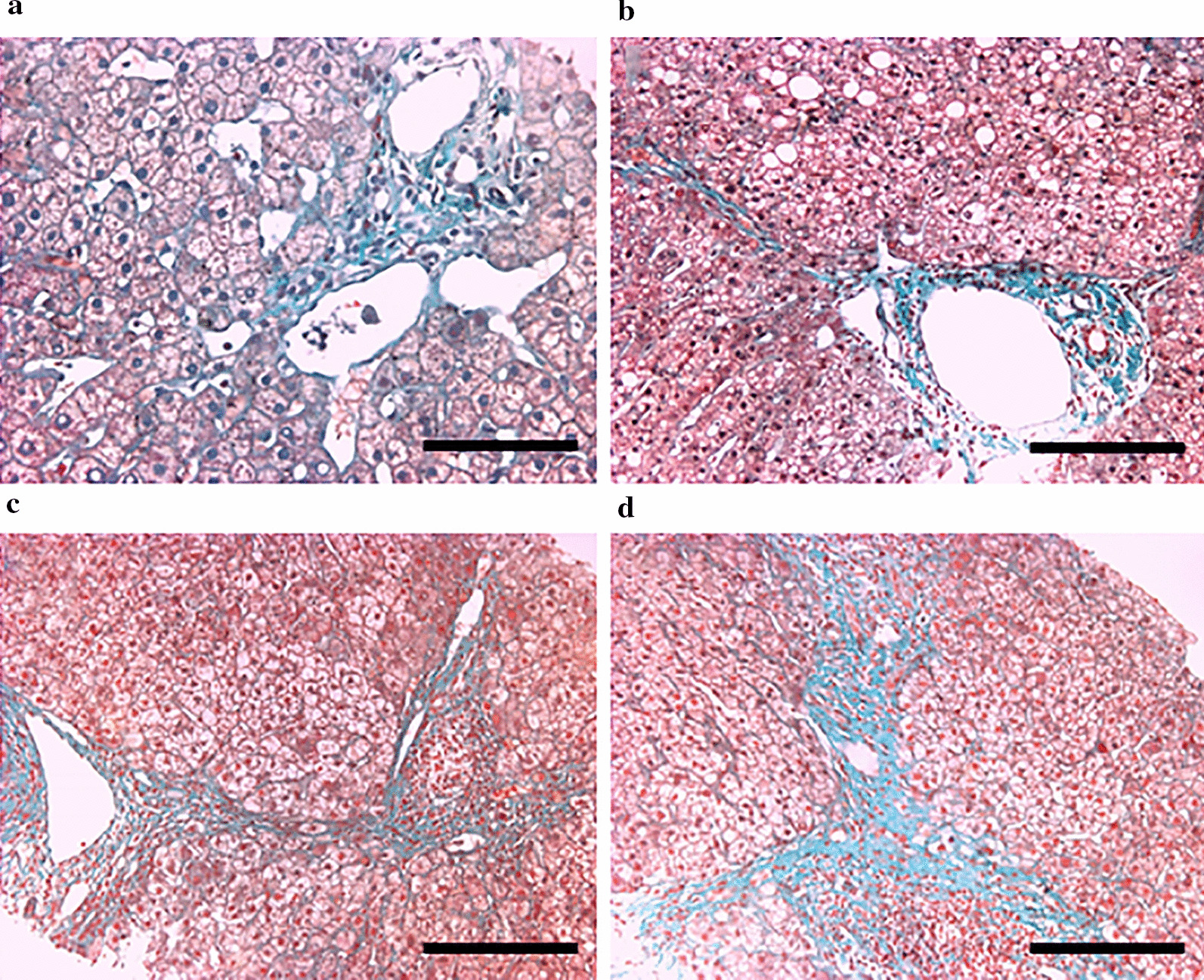
Histological H&E/Masson staining of the livers of monkeys with liver fibrosis at stages S1 (**a**), S2 (**b**), S3 (**c**) and S4 (**d**). Scale bar = 100 μm (Masson × 100)

Male healthy cynomolgus monkeys (n = 30) were used for the establishment of liver fibrosis models. Among them, eight monkeys died during the treatment process, while 22 survived and were able to establish the liver fibrosis model successfully. Therefore, the success rate for model establishment was calculated to be 73.3%. In these surviving monkeys, all of them progressed into the S1 stage of liver fibrosis (G0 ~ 1S1). The livers of these animals displayed complete hepatic lobule structure, loose cytoplasm of the liver cells in the lobule, hyperplasia of the fibrous tissue in the portal area, and infiltration of lymphocytes without the formation of a fibrous septum (Fig. 2a). Twenty monkeys progressed into the S2 stage (G1 ~ 2S2), in which, the liver tissues showed histological liver cell swelling, cytoplasmic loosening, fibrosis around the portal areas, and the formation of small amounts of fibrous septa with a still existing lobular structure (Fig. 2b). Seventeen monkeys progressed into stage S3 (G2 ~ 3S3). In the livers of monkeys in the S3 stage, the portal areas were widened, fibrous tissue was obviously proliferated, a large number of fibrous septa formed, the hepatic lobules were segmented to form pseudolobules, and the lobular structures were disorganized, but no cirrhosis formed (Fig. 2c). Fifteen monkeys progressed into the S4 stage (G3 ~ 4S4). Extensive destruction of the liver parenchyma, proliferation of diffuse fibrous tissues, and hepatic lobules segmentation to form pseudolobules were observed in the liver tissues of these monkeys (Fig. 2d). After 20 weeks of treatment, 15 monkeys developed complete pathology of liver fibrosis.

### Comparison of MR-PWI results of liver fibrosis in different stages

The cynomolgus monkeys that died during modeling (n = 8) or failed to develop into early liver cirrhosis (n = 7) were excluded from the investigation. The remaining monkeys with complete development of liver fibrosis (n = 15) were used for MR-PWI imaging and the detection indicators were compared. The results showed that the PWI quantitative parameters of cynomolgus monkeys, including Ktrans, Kep, and Vp, decreased with the progression of liver fibrosis (Table 1). In comparison with normal liver tissue (S0), the Ktrans and Kep of livers in all liver fibrosis stages (S1–S4) were significantly lower (P < 0.01). In the severe (S3 and S4), mild (S1), and moderate (S2) liver fibrosis stages, the Ktrans and Kep were also found to be significantly different (P < 0.01). However, no significant difference was observed between stages S1 and S2, and stages S3 and S4. With the development of liver fibrosis, HPI was found to gradually increase and showed significant differences between each two groups in stage S0–S4 (P < 0.01). Ve was also found to gradually increase, while there was no significant difference between these groups (P > 0.01). Spearman rank correlation analysis clearly indicated that Ktrans and Kep were negatively correlated with the stages of liver fibrosis severity (r_s_ = -0.875 for Ktrans and -0.797 for Kep, P < 0.01), while HPI had a high positive correlation with the stages of liver fibrosis (r_s_ = 0.959, P < 0.01).

**Table 1:** Comparison of the quantitative parameters of MR-PWI in different stages of liver fibrosis in cynomolgus monkeys (mean ± standard deviation)

Group	Case	Ktrans (ml/min)	Kep (ml/min)	Ve (ml/ml)	Vp (ml/ml)	HPI
Stage S0	15	0.584 ± 0.044	2.565 ± 0.482	0.226 ± 0.025	0.269 ± 0.036	0.244 ± 0.022
Stage S1	15	0.527 ± 0.038	2.199 ± 0.307	0.231 ± 0.029	0.263 ± 0.029	0.317 ± 0.035
Stage S2	15	0.479 ± 0.035	1.897 ± 0.301	0.236 ± 0.030	0.258 ± 0.022	0.421 ± 0.046
Stage S3	15	0.432 ± 0.032	1.524 ± 0.174	0.243 ± 0.032	0.251 ± 0.023	0.546 ± 0.043
Stage S4	15	0.377 ± 0.031	1.232 ± 0.130	0.246 ± 0.036	0.247 ± 0.021	0.651 ± 0.058
*F* Value		685.228	99.718	2.183	1.457	839.883
*P* Value		0.000	0.000	0.096	0.147	0.000

The semi-quantitative parameters of MR-PWI in different stages of liver fibrosis in cynomolgus monkeys were then analyzed. The TTP was gradually prolonged with the progression of liver fibrosis (Table 2), and the differences between each two groups were statistically significant (P < 0.01). MAX conc, MAX slope, and AUC were found to decrease gradually with the development of liver fibrosis. There was a significant difference between stages S3–S4 and S0–S2 (P < 0.01). No statistical difference was observed between the remaining groups. Spearman rank correlation analysis indicated that the TTP was highly positively correlated with the pathological stages of liver fibrosis (r_s_ = 0.921, P < 0.01), while the MAX conc, MAX slope, and AUC were negatively correlated with the pathological stages of liver fibrosis (r_s_ = −0.424 for MAX conc, −0.683 for MAX slope, and −0.616 for AUC; P < 0.01 for all).

### Diagnostic efficacy evaluation of MR-PWI quantitative parameters in early cirrhosis

**Fig. 3:**
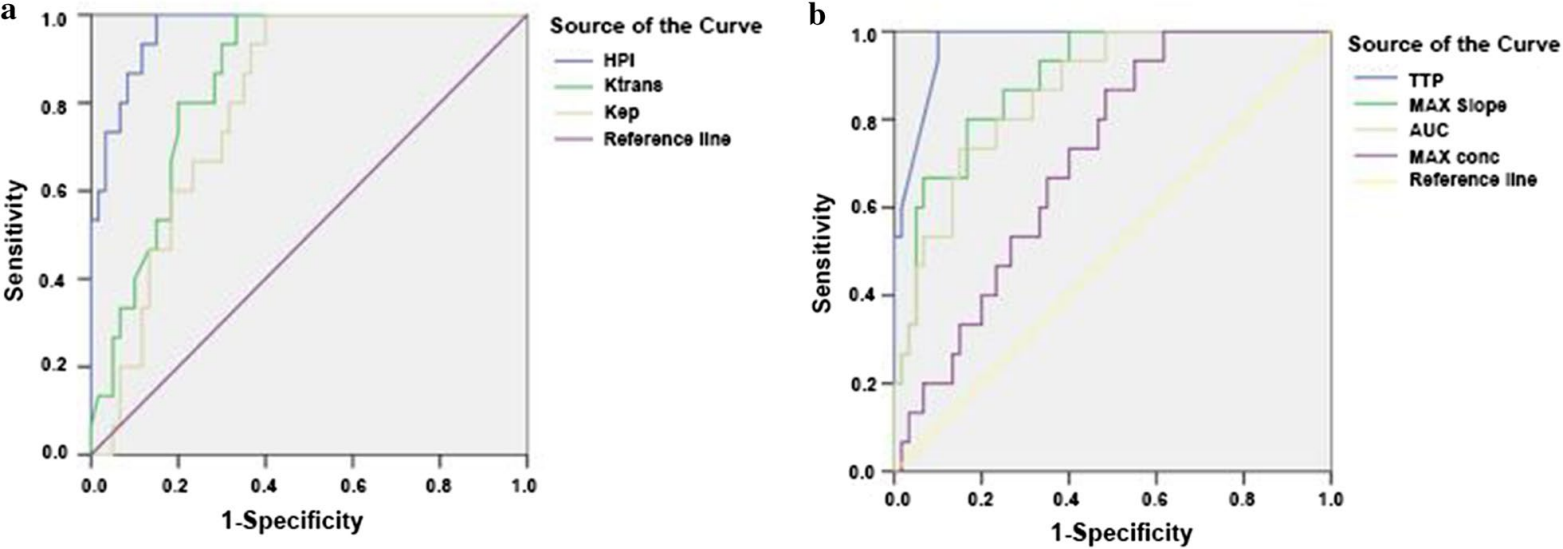
**a** Comparison of ROC curve analysis of HPI, Ktrans, and Kep for the diagnosis of early liver cirrhosis. **b** Comparison of ROC curve analysis of TTP, MAX Slope, AUC, and MAX conc for the diagnosis of early liver cirrhosis

In this study, ROC curves were used to evaluate the diagnostic efficacy of MR-PWI quantitative and semi-quantitative parameters in early cirrhosis. The areas under the ROC curve of MR-PWI quantitative parameters gradually decreased in the following order: HPI > Ktrans > Kep (Fig. 3a and Table 3). HPI had both the highest sensitivity and specificity with a Youden index (most diagnostic quantitative index) as high as 0.905. The sensitivity and specificity of Kep were very low, and thus the diagnostic efficiency was the worst. The optimal critical points (thresholds) of HPI for the diagnosis of liver fibrosis of different degrees were higher than 0.291, 0.376, 0.503, and 0.590 in stages S1, S2, S3, and S4, respectively (Table 4). For the most effective diagnosis, both the sensitivity and specificity were very high in stage S4.

**Table 2:** Comparison of the semi-quantitative parameters of MR-PWI in different stages of liver fibrosis in cynomolgus monkeys (mean ± standard deviation)

Group	Case	TTP (min)	MAXconc (mmol)	MAX slope (mmol/min)	AUC
Stage S0	15	1.292 ± 0.347	0.792 ± 0.229	1.913 ± 0.378	2.891 ± 0.447
Stage S1	15	1.935 ± 0.396	0.759 ± 0.224	1.852 ± 0.341	2.759 ± 0.368
Stage S2	15	2.552 ± 0.455	0.738 ± 0.242	1.729 ± 0.303	2.714 ± 0.351
Stage S3	15	3.363 ± 0.448	0.577 ± 0.174	1.324 ± 0.291	2.351 ± 0.291
Stage S4	15	4.169 ± 0.474	0.550 ± 0.139	1.115 ± 0.276	2.147 ± 0.282
*F *Value		11.349E3	22.451	100.371	53.202
*P* Value		0.000	0.000	0.000	0.000

The areas under the ROC curve of MR-PWI semi-quantitative parameters gradually decreased in the following order: TTP > MAX slope > AUC > MAX conc (Fig. 3b and Table 5). The TTP had the largest Youden index (0.85) and was the best diagnostic detection index. The optimal critical points (thresholds) of TTP for the diagnosis of liver fibrosis in different stages were higher than 1.714 min, 2.141 min, 2.852 min, and 3.595 min in stages S1, S2, S3, and S4, respectively (Table 6). The optimal critical points obtained by ROC curve analysis were used to diagnose liver fibrosis of different stages, and their sensitivity and specificity could be calculated. Both the highest sensitivity (0.933) and specificity (0.917) was found in stage S4.

**Table 3:** ROC curve analysis of MR-PWI quantitative parameters for early cirrhosis in cynomolgus monkeys

Parameters	AUC	Critical points	Sensitivity	Specificity	P value
Ktrans	0.853	0.395	0.867	0.774	0.000
Kep	0.799	1.361	0.796	0.719	0.000
HPI	0.967	0.590	0.974	0.931	0.000

**Table 4:** Optimal critical points (threshold) of HPI for the diagnosis of liver fibrosis in different stages

Stage	Thresholds	Sensitivity	Sensitivity	P value
Stage S1	≥ 0.291	0.957	0.895	0.000
Stage S2	≥ 0.376	0.938	0.847	0.000
Stage S3	≥ 0.503	0.944	0.913	0.000
Stage S4	≥ 0.590	0.974	0.927	0.000

**Table 5:** ROC curve analysis of MR-PWI semi-quantitative parameters for early cirrhosis in cynomolgus monkeys

Parameters	AUC	Critical points	Sensitivity	Specificity	P value
TTP	0.973	≥ 3.595	0.933	0.917	0.000
MAX conc	0.701	≤ 0.645	0.733	0.633	0.000
MAX Slope	0.859	≤ 1.422	0.867	0.751	0.000
AUC	0.827	≤ 2.371	0.801	0.717	0.000

**Table 6:** Optimal critical points (threshold) of TTP for the diagnosis of liver fibrosis in different stages

Stage	Thresholds	Sensitivity	Specificity	*P* value
Stage S1	≥ 1.714	0.917	0.800	0.000
Stage S2	≥ 2.141	0.925	0.833	0.000
Stage S3	≥ 2.852	0.928	0.911	0.000
Stage S4	≥ 3.595	0.933	0.917	0.000

## Discussion

### Establishment of a liver fibrosis model in cynomolgus monkeys and the clinical significance

Liver fibrosis is an important pathological feature and a reversible intermediate development stage for many chronic liver diseases. However, liver fibrosis without effective treatment leads to liver cirrhosis and even cancer. Therefore, it is extremely important to diagnose and treat liver fibrosis at the reversible early stages of cirrhosis, which is a major focus of current medical attention. Biopsies are a commonly used method for the detection of liver fibrosis. Because of the invasive nature of liver biopsy, it is impracticable to observe dynamically the progression of liver fibrosis in the clinic. To explore the effective strategies for the prevention and treatment of liver fibrosis, establishing a complete liver fibrosis animal model that is similar to humans with all developmental stages (S0-S4) is very important. Cynomolgus monkeys are a class of higher animals, and their physiological functions are closer to humans than other lower animals. Thus, they are often considered as the best model for human physiological studies. Previous studies have shown that the pathological characteristics of CCl_4_-induced liver fibrosis of different degrees (stages S1–S4) in cynomolgus monkeys are completely consistent with those of human liver fibrosis [25, 29]. The pathological grades of this liver fibrosis model are positively correlated with the pathological stage, which is similar to the pathological characteristics of human liver fibrosis. In addition, the blood perfusion parameters and variation tendency of serum markers and portal vein free pressure in the liver fibrosis model of cynomolgus monkeys are strikingly similar to those of humans, indicating the superiority of cynomolgus monkeys over other lower animals. As such, CCl_4_-induced liver fibrosis models in cynomolgus monkeys can provide an excellent experimental platform for the prevention and treatment of chronic liver diseases.

### The changes of MR-PWI parameters in liver fibrosis of different stages in cynomolgus monkeys

Since the general morphological changes of liver fibrosis are not prominent, image diagnosis of liver fibrosis is always difficult. Conventional biomedical imaging technologies such as ultrasound, computed tomography and magnetic resonance often fail to diagnose early-stage liver fibrosis [30, 31]. In the clinic, one of the major current methods for detection is invasive liver biopsy, which is unfavorable for the dynamic monitoring of liver fibrosis and the evaluation of therapeutic efficacy. Development of noninvasive methods for the early diagnosis of liver fibrosis and cirrhosis is very important and has become a hot topic of study in the medical field. TE, ARFI, MRE, MR-PWI, magnetic resonance sepectroscopy (MRS), diffusion-weighted imaging (DWI) and some other emerging technologies have become the current imaging research hotspots for non-invasive evaluation of liver fibrosis, and all of them have shown different degrees of diagnostic efficacy. TE and ARFI are able to diagnose severe liver fibrosis and cirrhosis, while failing to accurately identify the staging of mild liver fibrosis [16]. TE also has the disadvantage of limited penetration depth that affects the diagnostic results of patients with obesity, ascites, anatomical abnormalities, and elevated central venous pressure. The measured values of liver stiffness will be interfered by liver tissue inflammation, edema, and fat change. MRS is an imaging technique that utilizes MR equipment to obtain the MRS information of biochemical substances in living subjects [28, 32, 33]. Although MRS has been widely used in clinical practices, diagnosis of liver fibrosis using MRS has been poorly reported. Moreover, the examination results of MRS cannot be absolutely quantified and thus fail to reflect the actual condition of diseases [33]. Therefore, it is difficult to use and promote MRS for clinical diagnosis. DWI that reflects the microscopic movement of water molecules can also be used to diagnose liver fibrosis stages [34, 35]. Bonekamp et al. claimed that DWI should be further evaluated as the content of non-invasive MR imaging of liver fibrosis [36]. The developed diffusion kurtosis imaging based on DWI has been conducive to identify the stages of liver fibrosis [37, 38], while the DWI evaluation of liver fibrosis has not achieved a widely recognized diagnostic standard. In contrast, MR-PWI as a non-invasive functional imaging technology based on hemodynamics has become breakthroughs in the field of imaging medicine, making it possible for the early diagnosis of liver fibrosis before the morphological changes of the liver tissue [39–42]. More importantly, MR-PWI has the advantage of no radiation damage, and therefore, shows great promise for clinical applications.

MR-PWI mainly relies on rapid MRI sequences to collect images continuously and repeatedly before and after intravenous injection of contrast agents, and then obtains a series of quantitative and semi-quantitative parameters through post-processing of the images. It is a functional imaging technique that can simultaneously reflect the changes of tissue and organ morphologies and blood perfusion information [43, 44]. MR-PWI has shown unique advantages for the diagnosis of tumors in different tissues [45, 46], while its use for liver fibrosis detection has been poorly reported. In this study, an autologous pairing method was adopted to compare the MR-PWI quantitative and semi-quantitative parameters of 15 cynomolgus monkeys with complete liver fibrosis development (stages S0–S4). This allowed a real comparison of MR-PWI quantitative and semi-quantitative parameters before and after the establishment of a liver fibrosis model in each cynomolgus monkey, and objectively revealed the changes of PWI perfusion parameter values with the degrees of liver fibrosis. This study will provide a more reliable theoretical and practical basis for clinical noninvasive monitoring of liver fibrosis using a MR-PWI technique.

Liver fibrosis can lead to changes in the liver parenchyma and vascular structures, resulting in changes of liver hemodynamics and perfusion information. Conventional imaging technologies often fail to reflect such subtle morphological changes, while PWI can detect abnormal perfusion information before liver morphological changes, and thus allows for the early diagnosis of liver fibrosis and cirrhosis. Liver fibrosis is found to be closely related to hepatic sinus capillaries. The normal hepatic sinus is a discontinuous capillary, which is conducive to material exchange between liver cells and blood. However, the foramina of the hepatic sinus endothelium gradually decreases and even disappears with the development of liver fibrosis, ultimately leading to hepatic sinus capillarization [47]. Hepatic sinus capillarization will result in changes to hepatic perfusion and hemodynamics [48].

This study demonstrated that the MR-PWI quantitative parameters including Ktrans and Kep of cynomolgus monkeys gradually decreased with the progression of liver fibrosis. This can not only be used to identify the stages of liver fibrosis (S1–S4) and normal liver tissue (S0), but can also be used to different stages S3–S4 (severe liver fibrosis), S1 (mild liver fibrosis), and S2 (moderate liver fibrosis) (P < 0.01), which is similar to the previous report [49]. This is most likely due to the reduced material exchange between liver cells and blood caused by hepatic sinus capillarization. In addition, the deposition of liver fibrosis collagen fibers in the hepatic sinus and periphery of the sinusium will also cause increased blood flow resistance for the hepatic sinus. The rates of contrast agents entering into the extracellular space of blood vessels and their reflux are reduced, leading to gradually decreasing Ktrans and Kep. The quantitative parameter of HPI gradually increased with the progression of liver fibrosis, and thus HPI was able to identify liver fibrosis and early cirrhosis of different stages (S1–S4) with a significant difference between each group. The mechanism was proposed based on our results. In stages S1–S2 (mild to moderate liver fibrosis), liver cells degenerated and swelled, hepatic blood sinusoids narrowed due to compression, and hepatic artery perfusion and portal vein return were obstructed by the proliferation of interstitial fibers, resulting in an obvious obstruction of the portal vein blood flow and increased HPI. In stages S3–S4 (severe liver fibrosis), the degrees of fibrosis were significantly increased, the hepatic lobule structure was disordered and even formed cirrhotic nodules, intrahepatic vascular bed areas were significantly reduced, the blood perfusion resistance increased, and the portal vein perfusion flow was reduced, which led to a compensatory increase of hepatic artery perfusion due to hepatic artery buffering effects. Hence, HPI was obviously increased.

The MR-PWI semi-quantitative parameter of TTP was found to increase gradually with the progression of liver fibrosis. In view of the abnormal hepatic blood flow caused by hepatic fibrous tissue hyperplasia and formations of pseudolobuli, the increased unpaired small arteries, hepatic sinus capillarization, and posterior sinus congestion led to vascular stenosis, occlusion, and decreased liver blood perfusion rates. Meanwhile, the deposition of collagen around the hepatic sinus and extravascular space hindered the diffusion of contrast agents. These pathological changes are aggravated with the progression of liver fibrosis; and thus, TPP was found to gradually increase and show significant differences in each stage of liver fibrosis. Our results were consistent with the study using rat liver fibrosis model conducted by Fan et al. [42]. They showed that TTP and mean transit time values were positively correlated with hepatic fibrosis stage via Pearson's correlation analysis. MAX slope is the maximum slope of the time signal curve between the beginning and peak of strengthening, which represents the changes in amplitude and speed of the tissue signal increase caused by the inflow of contrast agents per unit time. MAX conc (maximum tissue concentration) and AUC can also reflect the perfusion information of tissues, but they are influenced by various factors such as the concentration of contrast agents, vascular permeability, and blood flow velocity. All semi-quantitative parameters including MAX conc, MAX slope, and AUC gradually decreased with the progression of liver fibrosis. However, a significant difference was only observed between the severe liver fibrosis stages (S3–S4) and stages S0–S2, indicating that these indicators had limited diagnostic value for the confirmation of the severity of liver fibrosis. In the S0, S1, and S2 stages, no significant differences were observed for MAX slope, MAX conc, and AUC. This may be due to the absence or minimal development of liver fibrosis in these stages, in which, the increase of portal vein blood flow resistance was relatively lower, and the hepatic artery could maintain total liver perfusion levels via autogenous regulation. However, the hepatic lobule structures were obviously damaged and early cirrhotic pseudolobuli formed in stages S3–S4, and thus the hepatic microcirculation resistance obviously increased, which led to unstable total hepatic perfusion levels. Thus, the MAX slope, MAX conc, and AUC in stages S3–S4 were significantly lower than those in normal and mildly fibrotic livers.

### Diagnostic efficacy evaluation of MR-PWI quantitative and semi-quantitative parameters in early cirrhosis

In this study, ROC curve analysis was used to evaluate the diagnostic efficacy of MR-PWI parameters in early cirrhosis. Our results indicated that TTP is the most important MR-PWI semi-quantitative parameter for the diagnosis of liver fibrosis with a Youden index of 0.85. Previous study showed that PWI perfusion parameters, especially TTP and mean transit time reflected the degree of hepatic fibrosis [42]. Since hepatic sinus capillarization and collagen depositions around the hepatic sinus and extravascular space can lead to a prolonged strengthening peak time of contrast agents, more severe liver fibrosis resulted in a longer TTP with a positive correlation. Moreover, TTP was minimally affected by other factors such as the contrast agents, indicating the stability and reliability of TTP. The poor diagnostic efficiency of MAX slope, AUC, and MAX conc are likely due to the influences of the concentration, dose, and injection rate of contrast agents, apart from the stages of liver fibrosis.

The investigations of MR-PWI quantitative parameters of liver fibrosis have been poorly reported so far. We herein adopted an exchange liver dual blood supply model to calculate the MR-PWI quantitative parameters including Ktrans, kep, Ve, Vp, and HPI. This method can overcome the limitations of the semi-quantitative parameter analysis, which was greatly affected by factors such as the signal intensity, injection dose, and rate of contrast agents, and scanning parameters. In view of the good repeatability, this can better reflect the blood perfusion information of different stages of liver fibrosis. Previous studies have shown that the MR-PWI parameters, Ktrans and Kep, cannot only identify liver fibrosis stages (S1–S4) and normal liver tissue (S0) but are also able to distinguish severe from mild and moderate liver fibrosis. However, it is difficult to confirm a consistent diagnosis threshold for the evaluation of liver fibrosis stages due to the inconsistency of magnetic resonance equipment scanning parameters and perfusion analysis software, individual subject differences, and the lack of convincing Ktrans and Kep reference standards. Therefore, the practical clinical applications of Ktrans and Kep are greatly limited by these concerns. In contrast, HPI has a reference standard in the clinic. The HPI of the normal population is about 0.2–0.3 with an average value of 0.25, and it is significantly different between the stages of liver fibrosis. Moreover, this index was hardly affected by the types and models of instruments, scanning parameters, and perfusion analysis software. In this study, we found that HPI had the highest diagnostic efficacy with a high Youden index of 0.905. Therefore, it is reasonable to believe that HPI is the most promising diagnostic quantitative index to evaluate liver fibrosis stages for MR-PWI.

HPI and TTP were hardly affected by some factors such as contrast agent concentration, dosage and injection rate. Moreover, HPI values will not be affected by equipment type and model, scanning parameters and perfusion analysis software. They are considered as the best quantitative and semi-quantitative detection indicators for PWI, respectively, and will play key roles in the future non-invasive assessment of liver fibrosis examination technology. In addition, since the correlation coefficient between the quantitative parameter HPI and the severity of liver fibrosis and the Youden index for diagnosing liver fibrosis staging are often higher than the semi-quantitative parameter TTP, it can be seen that the PWI quantitative parameters has a higher diagnostic efficacy than the semi-quantitative parameters. Quantitative parameters are suggested to be used for analysis and diagnosis of liver fibrosis. Therefore, it is crucial to consider the possibility of early-stage liver cirrhosis if the HPI is higher than 0.590 or TTP is longer than 3.595 min. In our continuative study, we will evaluate the feasibility of diagnosis of early-stage liver fibrosis in clinical patients using this non-invasive method.

## Conclusions

In summary, we established a liver fibrosis model in cynomolgus monkeys that was used to analyze the MR-PWI quantitative (Ktrans, Kep, Ve, Vp, HPI) and semi-quantitative parameters (TTP, Max Conc, Max slope, AUC) in different stages of liver fibrosis. These parameters changed with the development of liver fibrosis, among which, HPI and TTP were the best detection indices for the quantitative and semi-quantitative evaluation of liver fibrosis, respectively. Although these promising results, the applications of HPI and TTP for the clinical research and diagnosis of liver fibrosis need to be further validated.

## Data Availability

The datasets used and/or analysed during the current study are available from the corresponding author on reasonable request.

## Abbreviations

TE: Transient elastograhy (TE)
ARFI: Acoustic radiation force impulse imaging
MRE: Magnetic resonance elastography
MRI: Magnetic resonance imaging
MR-PWI: Magnetic resonance-perfusion weighted imaging
HE: Hematoxylin–eosin
Gd-DTPA: Gadolinium-diethylenetriamine penta-acetic acid
EES: Extravascular extracellular space
TTP: Time to peak
MAX conc: Maximum concentration
MAX slope: Maximum slope
AUC: Area under the concentration–time curve
ROC: Receiver operating characteristic
ROI: Region of interest
SD: Standard deviation
MRS: Magnetic resonance sepectroscopy
DWI: Diffusion-weighted imaging
